# Evaluation of an enzyme immunoassay and immunodiffusion for detection of anti‐*Histoplasma* antibodies in serum from cats and dogs

**DOI:** 10.1111/jvim.16726

**Published:** 2023-04-27

**Authors:** Rebecca Tims, Andrew S. Hanzlicek, Laura Nafe, Michelle M. Durkin, Jennifer Smith‐Davis, L. Joseph Wheat

**Affiliations:** ^1^ Veterinary Specialists of North Texas Fort Worth Texas USA; ^2^ Department of Veterinary Clinical Sciences Oklahoma State University Stillwater Oklahoma USA; ^3^ MiraVista Diagnostics Indianapolis Indiana USA; ^4^ Department of Veterinary Medicine and Surgery University of Missouri Columbia Missouri USA

**Keywords:** canine, feline, fungal infection, histoplasmosis, invasive fungal infection, serology

## Abstract

**Background:**

*Histoplasma* antigen and anti‐*Histoplasma* antibody detection are used to support the diagnosis of histoplasmosis. There is a paucity of published data on antibody assays.

**Objectives:**

Our primary hypothesis was that anti‐*Histoplasma* immunoglobulin G (IgG) antibody detection using enzyme immunoassay (EIA) will be more sensitive as compared to immunodiffusion (ID).

**Animals:**

Thirty‐seven cats and 22 dogs with proven or probable histoplasmosis; 157 negative control animals.

**Methods:**

Residual stored sera were tested for anti‐*Histoplasma* antibodies using EIA and ID. Results of urine antigen EIA were reviewed retrospectively. Diagnostic sensitivity was calculated for all three assays and compared between immunoglobulin G (IgG) EIA and ID. The diagnostic sensitivity of urine antigen EIA and IgG EIA, interpreted in parallel, was reported.

**Results:**

Sensitivity of IgG EIA was 30/37 (81.1%; 95% confidence interval [CI], 68.5%‐93.4%) in cats and 17/22 (77.3%; 95% CI, 59.8%‐94.8%) in dogs. Diagnostic sensitivity of ID was 0/37 (0%; 95% CI, 0%‐9.5%) in cats and 3/22 (13.6%; 95% CI, 0%‐28.0%) in dogs. Immunoglobulin G EIA was positive in all animals (2 cats and 2 dogs) with histoplasmosis but without detectable antigen in urine. Diagnostic specificity of IgG EIA was 18/19 (94.7%; 95% CI, 74.0%‐99.9%) in cats and 128/138 (92.8%; 95% CI, 87.1%‐96.5%) in dogs.

**Conclusion and Clinical Importance:**

Antibody detection by EIA can be used to support the diagnosis of histoplasmosis in cats and dogs. Immunodiffusion has an unacceptably low diagnostic sensitivity and is not recommended.

**Abbreviations**
EIA: enzyme immunoassay
EU: enzyme immunoassay units
GM: galactomannan
ID: agar gel immunodiffusion
IFI: invasive fungal infection
IgG: immunoglobulin G

## 1 INTRODUCTION

Histoplasmosis is an enzootic invasive fungal infection (IFI) of mammals worldwide. Veterinary species most commonly infected include domestic cats and dogs. After inhalation of microconidia found in soil, disease can be localized to the respiratory tract or disseminate via blood or lymphatics. After dissemination, multisystemic disease is most common, but disease can be localized to any organ.^1^, ^2^ Affected sites include bones, joints, gastrointestinal (GI) tract, eyes, or skin.^1^, ^2^, ^3^, ^4^

Diagnosis can be made by finding *Histoplasma* yeast organisms in tissue or body fluid samples, but doing so is not possible in some cases. Failure to identify the organism could be a result of disease in anatomic locations that preclude safe sampling or samples that contain low numbers of organisms. Culture of *Histoplasma* from affected tissues or body fluids is also confirmatory, but it is uncommonly used clinically, because turnaround time can be long (≥4 weeks).^5^ Because of these limitations, non‐culture‐based biomarkers often are used. These include *Histoplasma* antigen or anti‐*Histoplasma* antibodies. A commercially available enzyme immunoassay (EIA) detecting *Histoplasma* antigen (MVista *Histoplasma* Quantitative Antigen EIA, MiraVista Diagnostics, Indianapolis, IN) in urine, has a diagnostic sensitivity of 89% to 95% in cats and dogs.^6^, ^7^, ^8^, ^9^ Although cross‐reactivity occurs with closely related fungal organisms occurs such as *Blastomyces*, the test remains clinically useful because specificity for fungal vs non‐fungal disease is 97% to 100%.^6^, ^7^, ^8^ *Histoplasma* antigen testing also is used for treatment monitoring because concentrations decrease with successful treatment and increase with disease relapse.^10^

For the approximately 5% to 10% of cats and dogs with histoplasmosis that do not have detectable *Histoplasma* antigen in urine, detection of anti‐*Histoplasma* antibodies might be clinically useful. Currently, commercially available tests are either an enzyme immunoassay (EIA) or immunodiffusion (ID). Multiple diagnostic service laboratories in the United States offer ID for detection of anti‐*Histoplasma* antibodies, some as part of so‐called “fungal panels.” Immunodiffusion utilizes a clear agarose gel with multiple wells in close proximity. Purified *Histoplasma* mycelial H and M antigens, a catalase and β‐glucosidase, respectively, are used. Both antigens are added to one well and patient serum and positive control serum are added to adjacent wells. After incubation, a visible immunoprecipitation line between patient serum and antigen wells is evidence of anti‐*Histoplasma* antibodies in patient serum. The only commercially available EIA uses proprietary *Histoplasma* antigens for IgG capture.

Although anti‐*Histoplasma* antibody tests have been used for over 70 years in veterinary medicine, there remains a paucity of published data regarding their diagnostic performance.^11^ Our primary objective was to describe the diagnostic performance of commercially available anti‐*Histoplasma* antibody detection by IgG EIA and ID in cats and dogs. Our hypothesis was that IgG EIA would have a significantly higher diagnostic sensitivity as compared with ID. A secondary objective was to describe the combined diagnostic performance of IgG EIA and *Histoplasma* antigen EIA testing of urine. This comparison was done to investigate the clinical utility of IgG EIA because antigen testing is well established in clinical practice.

## 2 MATERIALS AND METHODS

Residual sera stored from cats and dogs enrolled in other clinical studies were used for antibody testing.^10^, ^12^ All samples were collected in accordance with study protocols approved by the respective Institutional Care and Use Committees. Pet‐owner signed consent was obtained at the time of study enrollment. Serum samples were from animals with histoplasmosis at the time of diagnosis and animals without histoplasmosis, either with an alternative diagnosis or healthy control animals.

### 2.1 Diagnosis and classification of histoplasmosis

Proven histoplasmosis required finding *Histoplasma* yeast organisms on cytopathology or histopathology by a board‐certified veterinary clinical pathologist or board‐certified veterinary anatomic pathologist, respectively. Probable histoplasmosis required consistent clinical findings and detectable *Histoplasma* antigen in urine using enzyme immunoassay (EIA). Disseminated histoplasmosis was defined as clinical evidence (e.g., laboratory test results, pathology, imaging results) of disease in any body system other than lung. Disseminated disease was further defined as disseminated localized or disseminated multisystemic. Disseminated localized disease was defined as disease of a single organ or organ system and associated lymph nodes (LN), other than the lung. Disseminated multisystemic disease was defined as clinical evidence of disease in ≥2 organ systems or finding *Histoplasma* organisms in blood (fungemia). Pulmonary histoplasmosis was defined as disease apparently localized to the lung and associated intrathoracic LN. Only sera collected from animals within 1 month of diagnosis were used to determine the diagnostic sensitivity of the anti‐*Histoplasma* antibody assays.

Data retrieved from medical record review for animals with histoplasmosis included signalment, clinical signs, and diagnostic test results (*Histoplasma* antigen EIA on urine, CBC, serum biochemistry, imaging studies, cytopathology and histopathology, and infectious disease and endocrine testing).

### 2.2 Control animals not diagnosed with histoplasmosis

Negative control sera were convenience samples from animals not diagnosed with histoplasmosis. These were primarily client‐owned animals from an area where histoplasmosis was enzootic along with a smaller number of sera from purpose‐bred research animals not expected to have been exposed to *Histoplasma*. An exclusion criterion for control animals was a diagnosis of histoplasmosis at any time, including before or after the time of sample collection. Medical record review for all control animals included coded diagnosis at all hospital visits. A more extensive review was performed on animals that were positive for anti‐*Histoplasma* antibodies on EIA, ID, or both. For these animals, medical record data extracted included signalment, diagnosis, routine laboratory test results, imaging results, and results of *Histoplasma* antigen EIA on urine, if performed.

### 2.3 *Histoplasma* antigen detection in urine using enzyme immunoassay

Results for *Histoplasma* antigen detection in urine (MVista *Histoplasma* Antigen EIA, MiraVista Diagnostics, Indianapolis, IN) at the time of diagnosis were reviewed for animals with histoplasmosis. The reportable concentration was 0.4 to 19.0 ng/mL. For statistical analysis, detection of *Histoplasma* antigen but below or above the limit of quantification was considered to be 0.4 and 19.0 ng/mL, respectively.

### 2.4 Anti‐*Histoplasma* IgG detection in serum using enzyme immunoassay

Commercially available laboratory‐developed EIAs (MVista *Histoplasma* Feline or Canine IgG EIA, MiraVista Diagnostics, Indianapolis, IN) with slight modifications to previously reported protocols were used.^13^, ^14^ Ninety‐six‐well microplates were coated with proprietary *Histoplasma* antigens and blocking buffer. Patient sera, standards, and controls were added to wells. After incubation at 37°C for 1 hour, wells were washed, and biotinylated anti‐canine or anti‐feline IgG was added to each well. The microplate was incubated at 37°C for 1 hour. The wells were washed, and streptavidin‐horse radish peroxidase conjugate was added to each well and then incubated at 37°C for 1 hour. The wells were washed, and chromogen solution containing peroxidase substrate was added and incubated at room temperature for 10 minutes. The enzymatic reaction was stopped by adding 2 N sulfuric acid. The plate was read at a dual wavelength of 450/620 nm. Immunoglobulin G antibody concentrations <8.0 enzyme immunoassay units (EU) were considered negative, 8.0 to 9.9 EU were considered indeterminant, and ≥10.0 EU were considered positive.

### 2.5 Anti‐*Histoplasma* antibody detection in serum using immunodiffusion

Testing was performed in accordance with reagent manufacturer instructions (Fungal Immunodiffusion Reagents, Meridian BioScience, Cincinnati, OH) based on the Ouchterlony double‐diffusion method.^15^, ^16^ In short, purified *Histoplasma* mycelial H and M antigens were added until full to a well on clear 0.9% agarose gel. Positive control serum and patient serum were added until full to adjacent wells, respectively. Gels were incubated at room temperature in airtight containers containing a wet gauze to avoid drying. Gels were inspected daily for up to 72 hours. Gels were read against a dark black background with a bright indirect light source. To be considered valid, two visible bands of immunoprecipitation, one for each antigen, must have been present between the control serum and antigen wells. The M antigen band is found closest to the patient serum well, whereas the H antigen band is found closest to the antigen well. To be interpreted as positive, one or two distinct bands must have been present between the patient serum well and the *Histoplasma* antigen well. In addition, the patient serum band(s) needed to make a smooth junction with the positive serum control band (full identity). If the positive control and patient serum bands crossed (partial identity), or if there was not a distinct visible line of precipitation between patient serum and antigen wells it was considered a negative test result.

### 2.6 Statistical analysis

Statistical analysis was performed using commercial software (Sigma Plot 14, Systat Software Inc., Palo Alto, CA). The Shapiro‐Wilk test was used to test for normality and antibody concentrations were non‐parametric (*P* < .001). Descriptive statistics were reported as median and range for continuous variables and frequency and percentage for nominal variables. Diagnostic sensitivity (true positive/[true positive + false negative]) was calculated for all three assays (IgG EIA, antibody ID, and antigen EIA) and diagnostic specificity (true negative/[true negative + false positive]) was calculated for the 2 antibody detection assays. Associated 95% confidence intervals (CI) were calculated using the exact Clopper‐Pearson method. The McNemar change test was used to compare the sensitivity between EIA with ID. The diagnostic sensitivity of the combination of antigen and IgG EIA interpreted in parallel was reported. Statistical significance was set as *P* ≤ .05.

## 3 RESULTS

### 3.1 Cats with histoplasmosis

Thirty‐seven cats with proven (n = 30) and probable (7) histoplasmosis were included. Median age was 5.0 years (range, 0.5‐17) and median body weight was 3.6 kg (range, 2.1‐7.8). There were 18 spayed females, 17 neutered males, and 1 each intact male and female. Domestic shorthair (n = 33) was the most common breed followed by domestic longhair (2), and Siamese (2). Diagnosis most often was proven on cytopathology (n = 27) followed by histopathology (3). *Histoplasma* organisms were found in one organ on cytopathology in 19 cats including LN (n = 7), spleen (4), skin (2), liver (1), kidney (1), lung (1), GI tract (rectal scrape, 1), bone and joint (1) and bone marrow (1). Organisms were found in multiple organs on cytopathology in 8 cats including liver and spleen (n = 4); spleen, LN, and skin (2); LN and skin (1); and spleen, LN, and joint fluid (1). Disease was proven by histopathology with organisms being found in the oral cavity (n = 2) and GI tract (1). Proven histoplasmosis was classified as disseminated multisystemic in 26 cats and disseminated localized in 4 cats. Localized disease involved the oral cavity (n = 2), GI tract (1), and bone and joints (1). No cat had proven histoplasmosis localized to the lung. For cats with probable histoplasmosis, pulmonary (n = 3) was the most common form, followed by disseminated localized (2) and disseminated multisystemic (2). Localized disease involved the eyes in both cats.

### 3.2 Dogs with histoplasmosis

Twenty‐two dogs with proven (n = 21) and probable (1) histoplasmosis were included. Median age was 5.0 years (range, 1‐13) and median body weight was 9.7 kg (range, 2.9‐53.5). There were 12 spayed females, 7 neutered males, 2 intact females, and 1 intact male. Dogs of mixed breeding (n = 7) were most common followed by miniature schnauzer (4), Siberian husky (2), Labrador retriever (2), and 1 each of great Dane, boxer, Jack Russell terrier, Boston terrier, beagle, Maltese, and miniature poodle. Diagnosis was proven by finding organisms on cytopathology. *Histoplasma* organisms were found in a single organ in 17 dogs including GI tract (rectal scrape, 8), blood (3), liver (2), abdominal effusion fluid (2), bone and joint (1), and skin (1). Organisms were found in multiple organs in 4 dogs including liver and LN (n = 2); liver, LN, and spleen (1); and, liver, blood, and abdominal effusion fluid (1). Disease was classified as disseminated multisystemic in 12 dogs and disseminated localized in 9 dogs. Localized disease involved the GI tract (n = 7), bone and joint (1), and skin (1). No dog had proven histoplasmosis localized to the lung. The dog with probable histoplasmosis (urine antigen, 1.4 ng/mL) had diffuse unstructured interstitial lung disease.

### 3.3 Cats with histoplasmosis: *Histoplasma* antigen and anti‐*Histoplasma* antibody testing

**FIGURE 1:**
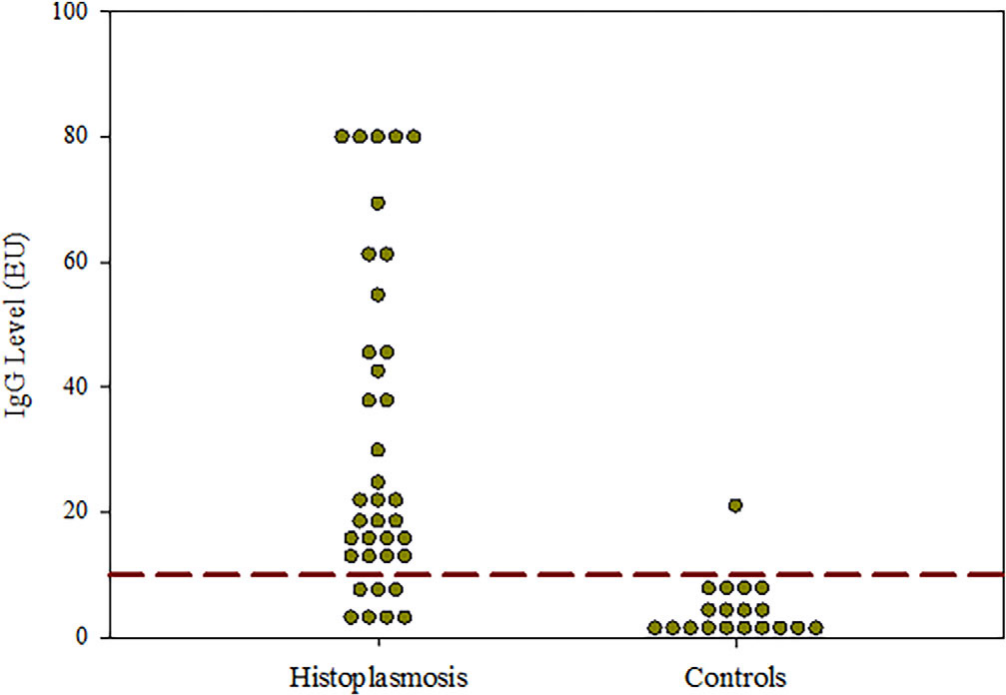
IgG EIA concentrations in serum in 37 cats with proven and probable histoplasmosis and 19 control cats without histoplasmosis. Each dot represents an individual cat's EIA result. The dashed line is the diagnostic cutoff (>10 EU).

Anti‐*Histoplasma* IgG EIA was positive in 24/30 (80.0%; 95% CI, 61.4%‐92.3%) cats with proven histoplasmosis (Tables 1 and 3). The median IgG antibody concentration was 23.2 EIA units (EU; range, 0‐80; Figure 1). All cats were negative for anti‐*Histoplasma* antibodies by ID. Urine was tested for *Histoplasma* antigen in all cats at the time of diagnosis and was positive in 28/30 (93.3%; 95% CI, 77.9%‐99.2%). The median antigen concentration was 4.2 ng/mL (range, 0‐19). Anti‐*Histoplasma* IgG EIA concentrations were >80 EU in both cats with no detectable *Histoplasma* antigen. Of these two cats, *Histoplasma* organisms were found on histopathology of an oral lesion in 1 and fine needle aspirate cytology of the kidneys in the second cat. Three cats (10.0%) had low (≤0.4 ng/mL) antigen concentrations and IgG EIA was positive in all three at 13.2, 19.6, and 29.9 EU. *Histoplasma* antigen was detected in all six cats with negative IgG EIA at 2.4, 2.9, 4.9, 7.3, 7.6, and 7.9 ng/mL.

**TABLE 1:** Diagnostic sensitivity of anti‐*Histoplasma* antibody detection in serum using ID or IgG EIA and *Histoplasma* antigen detection in urine using EIA in 37 cats with proven or probable histoplasmosis.

Test(s)	Positive tests/animals with histoplasmosis (sensitivity, 95% CI)		
	Proven	Probable	Total
Antibody ID	0/30 (0%, 0%‐11.6%)	0/7 (0%, 0%‐41.0%)	0/37 (0, 0%‐9.5%)
IgG EIA	24/30 (80.0%, 61.4%‐92.3%)	6/7 (85.7%, 42.1%‐99.6%)	30/37 (81.1%, 64.8%‐92.0%)
Antigen EIA	28/30 (93.3%, 77.9%‐99.2%)	7/7 (100%, 59.0%‐100%)	35/37 (94.6%, 81.8%‐100%)
IgG or Antigen EIA	30/30 (100%, 88.4%‐100%)	7/7 (100%, 59.0%‐100%)	37/37 (100%, 90.5%‐100%)

Abbreviations: EIA, enzyme immunoassay; ID, immunodiffusion.

Antigen was detected in 7/7 (100%; 95% CI, 59.0%‐100%) cats with probable histoplasmosis with median concentration of 7.0 ng/mL (range, 0.4‐15.8). Anti‐*Histoplasma* IgG EIA was positive in 6/7 (85.7%; 95% CI, 42.1%‐99.6%) with median IgG concentration of 15.0 EU (range, 2.4‐37.0). The diagnostic sensitivity of IgG EIA in all cats with histoplasmosis (proven and probable) was 30/37 (81.1%; 95% CI, 68.5%‐93.4%). All cats with probable histoplasmosis were negative for ID antibodies.

### 3.4 Dogs with Histoplasmosis—*Histoplasma* antigen and anti‐*Histoplasma* antibody testing

**FIGURE 2:**
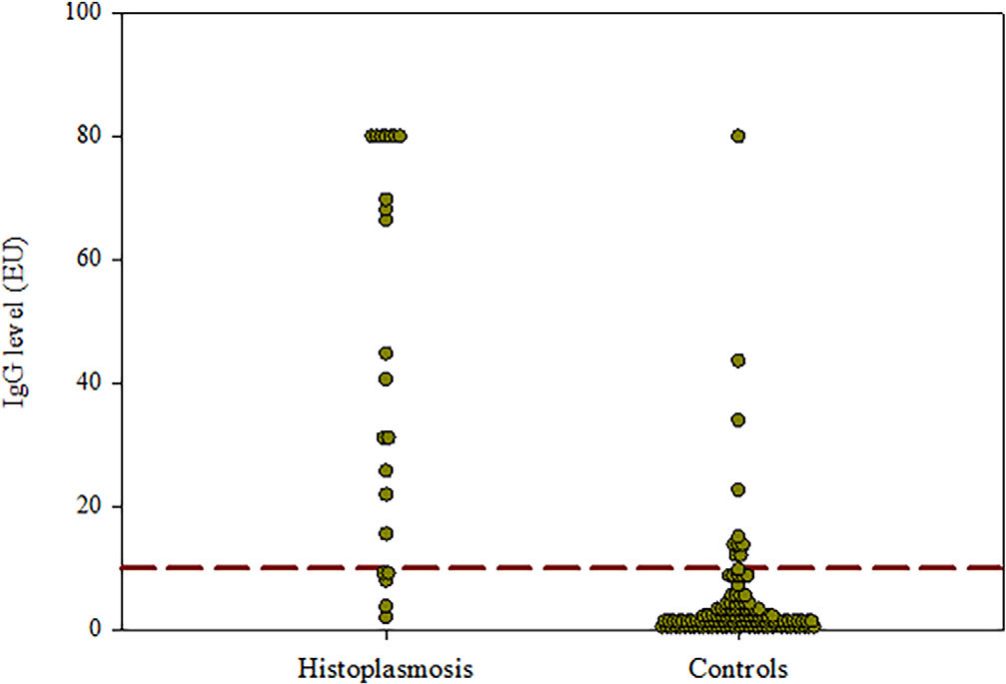
IgG EIA concentrations in serum in 22 dogs with proven and probable histoplasmosis and 120 control dogs without histoplasmosis. Each dot represents an individual dog's EIA result. The dashed line is the diagnostic cutoff (>10 EU).

Anti‐*Histoplasma* IgG EIA was positive in 17/21 (81.0%; 95% CI: 58.1%‐94.6%) dogs with proven histoplasmosis (Tables 2 and 3). The median IgG EIA concentration was 44.8 EU (range, 3.8 to >80). Of the dogs with negative IgG EIA, 2/4 had concentrations in the indeterminant range at 9.1 EU each. Samples from later time points were not available for either dog (Figure 2). Three dogs (14.3%; 95% CI, 3.0%‐36.3%) had detectable anti‐*Histoplasma* antibodies by ID. Two dogs had antibodies to M antigen and 1 to H antigen. In all three dogs, the IgG EIA concentrations were >80 EU. Diagnostic sensitivity of antibody detection by EIA was significantly higher as compared with ID (*P* < .001). *Histoplasma* antigen was detected in urine in 19/21 (90.4%; 95% CI: 69.6%‐98.8%) dogs. Median antigen concentration was 6.9 ng/mL (range, 0 to >19). Anti‐*Histoplasma* IgG EIA concentrations were 21.9 and >80 EU, respectively, in the two dogs with no detectable *Histoplasma* antigen. One of these dogs with disseminated multisystemic disease had organisms found on rectal scrape and the other with disseminated localized disease had organisms found on skin cytology. Three (14.3%) dogs had low (≤0.4 ng/mL) antigen concentrations, and IgG EIA was positive in all three at 44.8, >80, and >80 EU, respectively. *Histoplasma* antigen was detected in urine in all four dogs with negative IgG EIA at 0.5, 1.2, 6.9, and 15.8 ng/mL, respectively. *Histoplasma* antigen was detected in urine at 1.4 ng/mL in the dog with probable pulmonary histoplasmosis. This dog was negative for anti‐*Histoplasma* antibodies by ID and IgG EIA. The diagnostic sensitivity of IgG EIA in all dogs with histoplasmosis (proven and probable) was 17/22 (77.3%; 95% CI, 59.8%‐94.8%).

**TABLE 2:** Diagnostic sensitivity of anti‐*Histoplasma* antibody detection in serum using ID or IgG EIA and *Histoplasma* antigen detection in urine using EIA in 22 dogs with proven or probable histoplasmosis.

Test(s)	Positive tests/animals with histoplasmosis (sensitivity, 95% CI)		
	Proven	Probable	Total
Antibody ID	3/21 (14.3%, 3.0%‐36.3%)	0/1 (0%, 0%‐97.5%)	3/22 (13.6%, 2.9%‐34.9%)
IgG EIA	17/21 (81.0%, 58.1%‐94.6%)	0/1 (0%, 0%‐97.5%)	17/22 (77.3%, 54.6%‐92.2%)
Antigen EIA	19/21 (90.5%, 69.6%‐98.8%)	1/1 (100%, 2.5%‐100%)	20/22 (91.0%, 70.8%‐98.9%)
IgG or Antigen EIA	21/21 (100%, 83.9%‐100%)	1/1 (100%, 2.5%‐100%)	22/22 (100%, 84.6%‐100%)

Abbreviations: EIA, enzyme immunoassay; ID, immunodiffusion.

**TABLE 3:** Diagnostic specificity of anti‐*Histoplasma* antibody detection in serum using ID or IgG EIA in 19 cats and 138 dogs without histoplasmosis.

Host species	Negative tests/animals without histoplasmosis	Specificity (95% CI)
Antibody ID		
Cat	19/19	100% (82.3‐100%)
Dog	136/138	98.6% (94.9‐99.8%)
IgG EIA		
Cat	18/19	94.7% (75.1‐99.9%)
Dog	128/138	92.8% (87.1‐96.5%)

Abbreviations: EIA, enzyme immunoassay; ID, immunodiffusion.

### 3.5 Cats without histoplasmosis—Anti‐*Histoplasma* antibody testing

Serum samples from 19 cats not diagnosed with histoplasmosis were tested for anti‐*Histoplasma* antibodies by ID and IgG EIA. All cats lived in an area where histoplasmosis was enzootic. One (5.3%; 95% CI, 0.13%‐26.0%) cat tested positive, resulting in a diagnostic specificity of 94.7% (95% CI, 74.0%‐99.9%). This cat had an IgG EIA concentration of 21.0 EU and was diagnosed with multicentric lymphoma by fine needle aspirate and cytopathology. Two other cats had indeterminant IgG EIA concentrations between 8 and 10 EU. None of these three cats had antigen testing performed. No cat tested positive for ID antibodies.

### 3.6 Dogs without histoplasmosis—Anti‐*Histoplasma* antibody testing

Sera from 138 dogs not diagnosed with histoplasmosis were tested for anti‐*Histoplasma* antibodies. These included 120 client‐owned dogs and 18 purpose‐bred research dogs. None of the purpose‐bred research dogs was positive on IgG EIA or ID. All client‐owned dogs lived in an area where histoplasmosis was enzootic. Ten (10/120, 8.3%; 95% CI, 4.1%‐14.8%) were positive for IgG EIA. The median IgG EIA concentration in these dogs was 14.6 EU (range, 11.6 to >80). Four dogs had concentrations >20 EU at 22.6, 34.0, 43.6, and >80 EU, respectively. Six additional dogs had indeterminant IgG EIA concentrations between 8 and 10 EU. The diagnostic specificity of anti‐*Histoplasma* IgG in all dogs without histoplasmosis was 128/138 (92.8%; 95% CI, 87.1%‐96.5%). Two dogs tested positive for anti‐*Histoplasma* ID antibodies. One of these dogs had IgG EIA >80 EU and 1 dog was negative on IgG EIA. Additional case details are available for dogs that were positive for IgG EIA or ID antibodies (Table S1). For the 109 client‐owned control dogs not positive on IgG EIA or ID, the primary diagnosis was classified as GI or liver or pancreatic (n = 25), neoplasia (20), neurologic (13), cardiovascular (10), endocrine (8), immune‐mediated (7), invasive fungal infection (IFI) not histoplasmosis (6), musculoskeletal (5), respiratory (4), urologic (4), ocular (1), and toxicity (1). Five control dogs were determined to be apparently healthy. The dogs with IFI (other than histoplasmosis) included disseminated blastomycosis (n = 2), nasal aspergillosis (2), disseminated aspergillosis (1), and disseminated coccidioidomycosis (1). One dog with disseminated blastomycosis had an IgG EIA concentration in the indeterminant range at 9.7 EU. All other dogs were negative on IgG EIA. All six dogs with IFI (not histoplasmosis) were negative for ID antibodies.

## 4 DISCUSSION

Our study provides diagnostic performance data for two commercially available anti‐*Histoplasma* antibody assays for cats and dogs. Most importantly, we showed that anti‐*Histoplasma* antibody detection by ID has unacceptably low diagnostic sensitivity. In addition, we showed that antibody detection by EIA provides an acceptable diagnostic performance for active histoplasmosis in animals living in an enzootic area. Finally, our findings support the clinical use of anti‐*Histoplasma* IgG EIA when *Histoplasma* antigen testing of urine is negative and clinical suspicion remains or for additional evidence of histoplasmosis when antigen concentrations in urine are low (<0.4 ng/mL).

Anti‐*Histoplasma* antibody detection by ID has been used for many decades to support the diagnosis of histoplasmosis in cats and dogs.^11^ It is offered commercially by several veterinary diagnostic laboratories, often as part of so‐called “fungal panels,” which include antibody detection by ID for multiple pathogens. Until our study, no published data was available regarding the diagnostic performance of ID for histoplasmosis. Our findings suggest that histoplasmosis should not be ruled out based on a negative ID antibody test. In fact, the diagnostic sensitivity is so low that the use of ID for histoplasmosis in cats and dogs cannot be recommended.

As in veterinary medicine, ID has long been used for anti‐*Histoplasma* antibody detection in human medicine. In one study the diagnostic sensitivity was 37% in people with acute pulmonary histoplasmosis.^13^ The sensitivity increased to 56% when people exposed during point‐source outbreaks also were included.^13^ In a separate study, sensitivity was 78% for people with disseminated or pulmonary histoplasmosis.^17^ One possible explanation of the difference between dogs and cats vs humans might be that the H and M antigens traditionally used in ID testing are not dominant immunogens in cats and dogs. If so, this situation is likely paired with a difference in analytical sensitivity between the test methods. In our study, the three dogs with histoplasmosis and detectable ID antibodies had high IgG EIA concentrations (>80 EU). Collectively, this finding suggests that anti‐*Histoplasma* antibody concentrations in cats and dogs often fall below the limit of detection of ID, either because the analytical sensitivity is lower than that of EIA, there is a weak humoral immune response to H and M antigens, or both.

In humans, concurrent immunosuppressive conditions can lead to a blunted humoral immune response and false negative ID antibody testing.^17^ This situation is most often encountered with disseminated histoplasmosis.^17^ Although disseminated disease is most common in cats and dogs, comorbidities causing immunosuppression are uncommonly identified and such a situation was considered an unlikely cause of low ID antibody detection in our study.^2^, ^9^, ^18^ Immunodiffusion has been used successfully to detect other fungal pathogens. Most notably, it is a common biomarker test used to support the diagnosis of coccidioidomycosis (Valley Fever).^19^, ^20^ For coccidioidomycosis, ID has a diagnostic accuracy similar to EIA with a reported sensitivity of 97% to 100% and 87% to 89% in cats and dogs, respectively.^19^, ^20^, ^21^, ^22^ Immunodiffusion also has been used to detect anti‐*Blastomyces* and anti‐*Aspergillus* antibodies in cats and dogs, although as for histoplasmosis, ID has a lower diagnostic sensitivity as compared to EIA.^14^, ^23^, ^24^

More animals with histoplasmosis had detectable antigen in urine as compared to positive IgG EIA. As such, antigen detection in urine should be the initial non‐invasive biomarker considered. The pooled average sensitivity of antigen detection in urine in published studies is 93% and 94%, for dogs and cats, respectively, meaning that 6% to 7% of animals will have false negative results.^6^, ^7^, ^8^, ^9^ All four animals in our study with histoplasmosis and no detectable antigen were positive on IgG EIA. Thus, when interpreted in parallel (either test is positive and then diagnostic for histoplasmosis), the combined diagnostic sensitivity was 100%. The finding of *Histoplasma* antigen concentrations (<0.4 ng/mL) in urine is a clinical challenge, because intuitively those close to the diagnostic cutoff are more likely to be false positive. Although this situation has not been well studied in veterinary species, it has been reported in humans.^25^ In our study, the seven animals (3 dogs and 4 cats) with histoplasmosis and low antigen concentrations all were positive on IgG EIA. Collectively, these findings support the use of combined antigen and IgG EIA testing. More specifically, IgG EIA should be considered when antigen testing is negative and clinical suspicion remains, or for additional evidence of histoplasmosis when urine antigen concentrations are low.

Our study had some limitations. The first is that all testing was performed in a single‐service laboratory. Diagnostic performance, even of the same test, is laboratory specific. Neither a commercially available United States Department of Agriculture‐licensed anti‐*Histoplasma* ID test kit nor reagents are available for the detection of antibodies in cats or dogs. United States Federal Drug Administration‐approved or cleared ID test materials intended for use with human sera are available from several manufacturers and test components potentially could be manufactured in‐house. In addition, analytical procedures even for the same test kit might differ among laboratories. Finally, the interpretation of ID testing is subjective, requiring visual inspection to identify a band of immunoprecipitation. All of the above variables notwithstanding, it is unlikely a large enough difference exists in the diagnostic performance of ID testing among veterinary laboratories to recommend its use for anti‐*Histoplasma* antibody detection in cats and dogs.

The second limitation is that negative control animals used to establish diagnostic specificity had inconsistent diagnostic evaluations. These differences could have led to the inclusion of a small number of animals with occult histoplasmosis. The participating hospital was located in an endemic area had clinicians that were very familiar with histoplasmosis. Even in an endemic area, the overall prevalence of histoplasmosis in the participating hospital patient population during the study period was only 0.14% for dogs and 2.3% for cats. The prevalence of histoplasmosis in the control group would be expected to be even lower because animals in this group had at least 1 hospital visit, and in some cases extensive diagnostic evaluations, without being diagnosed with histoplasmosis. Collectively, these considerations suggest that inclusion of animals with occult histoplasmosis in the control group, if it did occur, was very uncommon. Albeit imperfect, our study provides an estimation of diagnostic specificity in an endemic area that will be useful for the practicing clinician. We decided to include all animals not diagnosed with histoplasmosis in the control group to avoid excluding animals without a definitive diagnosis but having overlapping clinical signs with histoplasmosis, which could have falsely inflated the reported specificity. For example, more extensive medical record reviews of the two control dogs with the highest IgG EIA concentrations could have been excluded because of overlapping clinical findings (Table S1). One dog had diffuse interstitial lung disease, pulmonary hypertension, and was receiving immunomodulatory medications for immune‐mediated neutropenia. This dog was also positive for anti‐*Histoplasma* ID antibodies. The second dog had tracheobronchial lymphadenopathy and a lytic vertebral lesion. Both dogs were negative for *Histoplasma* antigen in urine, but neither was tested for anti‐*Histoplasma* antibodies as part of the diagnostic investigation.

In conclusion, anti‐*Histoplasma* antibody detection by EIA is clinically useful, especially when combined with antigen detection. Immunodiffusion has an unacceptably low diagnostic sensitivity and its use for anti‐*Histoplasma* antibody detection in cats and dogs is not recommended.

## CONFLICT OF INTEREST DECLARATION

Andrew Hanzlicek, Michelle Durkin, Jennifer Smith‐Davis, and L. Joseph Wheat are employed by MiraVista Diagnostics which commercially offers the antigen and antibody detection tests used in this study. No other authors declare a conflict of interest.

## OFF‐LABEL ANTIMICROBIAL DECLARATION

Authors declare no off‐label use of antimicrobials.

## INSTITUTIONAL ANIMAL CARE AND USE COMMITTEE (IACUC) OR OTHER APPROVAL DECLARATION

Authors declare no IACUC or other approval was needed.

## HUMAN ETHICS APPROVAL DECLARATION

Authors declare human ethics approval was not needed for this study.

## Supporting information


**Appendix S1.** Supporting Information.
