# Supplementary material for: Evaluation of an enzyme immunoassay and immunodiffusion for detection of anti‐Histoplasma antibodies in serum from cats and dogs
Source: J Vet Intern Med. 2023 Apr 27;37(3):1007–14. doi: 10.1111/jvim.16726 (PMC10229331; doi:10.1111/jvim.16726)
Supplement: Supplementary file 1 — Appendix S1. Supporting Information. [file JVIM-37-1007-s001.pdf]

Supplementary Table 1. Clinical findings in animals with positive anti-*Histoplasma* serum IgG EIA or ID but not diagnosed with histoplasmosis.

MBD, mixed breed dog; EIA, enzyme immunoassay; ID, immunodiffusion; GM, galactomannan antigen (urine); TXR, thoracic radiographs; AUS, abdominal ultrasound; ND, none done

Breed	Age (y)	Active Diagnoses at hospital visit	Pertinent Clinical findings	IgG EIA (EU)	ID	Antigen EIA urine (ng/ml)
Labrador retriever	12	Interstitial lung disease; pulmonary hypertension	TXR, diffuse interstitial pattern: receiving azathioprine and prednisone for immune mediated neutropenia	>80	Positive	Negative
Boston terrier	10.5	Protein losing nephropathy; systemic hypertension; suspect hyperadrenocorticism	Azotemia, systemic hypertension, proteinuria	43.6	Negative	ND
Pointer	9	Multisystemic inflammatory disease undiagnosed	TXR, tracheobronchial lymphadenopathy, diffuse bronchointerstitial pattern; cytology of intraluminal bronchial nodule showed pyogranulomatous inflammation	34	Negative	Negative
MBD	12	Metastatic pheochromocytoma; babesiosis	Small *Babesia sp*. seen on blood smear; CBC, hemolytic anemia and thrombocytopenia; pheochromocytoma and metastasis to liver confirmed on histopathology	22.6	Negative	ND
Labrador retriever	9	Steroid responsive meningitis; immune mediated polyarthritis	Neutrophilic pleocytosis and suppurative inflammation in joint fluid on cytology.	15.1	Negative	ND
Alaskan malamute	12	Acute pancreatitis, hepatopathy undiagnosed; acute kidney injury; GI hemorrhage	TXR, normal; AUS, diffusely heterogeneous liver, hypoechoic pancreas with surrounding hyperechoic mesentery, small volume effusion; mild suppurative inflammation on liver cytology	14.1	Negative	ND
American Staffordshire terrier	7.5	Flea allergy dermatitis	Diagnosis based on physical exam and historical data	13.7	Negative	ND
Pekingese	13	Epilepsy; non-functional adrenal tumor	AUS, adrenal mass; Normal low dose dexamethasone suppression test	13.5	Negative	ND
MBD	11	Hyperadrenocorticism; cystic endometrial hyperplasia	AUS, bilaterally enlarged adrenals, ovarian cysts, uterine wall thickened with multiple anechoic cysts.	12.5	Negative	ND
Miniature Schnauzer	7	Sterile nodular panniculitis; diabetes mellitus	Sub-cutaneous ulcerative masses, histopathology showed pyogranulomatous inflammation, negative staining for fungi and Mycobacterium, responded to immunomodulatory treatment.	11.6	Negative	ND
Boxer	7.5	T-cell lymphoma	Lymphoma diagnosed on FNA cytology from multiple peripheral lymph nodes	Negative	Positive	ND
Domestic shorthair	11.5	Lymphoma	Small intestinal and nasopharyngeal lymphoma diagnosed on histopathology	21.0	Negative	ND
